# Elements of Chemistry, Including the Applications of the Science to the Arts

**Published:** 1843-09-30

**Authors:** 


*Elements of Chemistry, including the Applications of the Science in the Arts. With numerous illustrations.* By Thomas Graham, F. R. S. L., and E. Professor of Chemistry in University College, London; President of the Chemical Society; Corresponding Member of the Royal Academies of Sciences of Berlin and Munich. *With Notes and Additions.* By Robert Bridges, M. D., Professor of General and Pharmaceutic Chemistry in the Philadelphia College of Pharmacy, and one of the Editors of the American Journal of Pharmacy. Philadelphia: Lea & Blanchard. 1843. 8vo. pp. 746.

The general principles of chemical philosophy have recently made so much progress, that a brief account of the present condition of that interesting and important branch of science would no doubt be acceptable to our readers. For some time past it has been our intention to offer a hasty sketch of the actual state of Chemistry, to note the chief advances it has made, and detail its more important discoveries. We had proposed to ourselves to seize the present occasion as the most fitting one, and, with the work of one who had done so much to advance the theory of the science, to enter at some length on the relation and discussion of the new views embodied in it. This pleasing task, however, we are at present obliged to forego, and postpone to another opportunity the concise review we had intended to give. Professor Graham justly ranks as the first of British philosophical chemists; no single individual in Great Britain, within the last fifteen years, has contributed so much to the advancement of Chemistry. His work is one of the most profound and comprehensive that has been published in any country; distinguished alike for the high order of mind which it shows, and the clear and concise manner in which all that it treats of is handled. To the advanced student, and as a book of reference, we can cordially recommend it. Many portions of it are, we fear, too abstruse for the ordinary student. We therefore could have wished the American Editor to have been somewhat less sparing of his annotations, and to have given us, in many places, somewhat more extended remarks and commentaries on many of the theoretical portions of the work—a task for which he was abundantly qualified, and which would materially have added to its value. It has undergone a faithful revision at his hands, and many judicious notes have been added. One part, only, of his labours are we disposed to regret. It is well known that the doctrine of compound radicles—that is, of combinations of two or more elements, which, in their chemical relations with other bodies, correspond with simple substances—has been extended by Professor Liebig to inorganic chemistry, and that the most hearty advocate of this view is Professor Graham, who has contributed largely to its more perfect elaboration. This binary theory of salts is at most, however, proposed in the present work as affording a simple and philosophical explanation of certain actions—as hypotheses which, for the present, at least, serve the purpose, until they are fully proven, or something more certain proposed in their stead. Now, in the chapter on the "Arrangement of the Elements of

Compounds," the American Editor, in lieu of adding any remarks of his own, illustrative or deprecatory, has placed without comment numerous extracts from Professor Hare's « Effort to refute the argument in favor of the existence in the Amphide Salts, of Radicals." We are sorry for this, for we think it will tend to obscure, in place of elucidating the subject under discussion, and at the same time give currency to illogical and unsound views. The learned professor has, we think, signally failed in grasping the bearings of the question, and has succeeded in enveloping it in an impenetrable mist.

